# Antibacterial evaluation silver of nanocomposite on *Staphylococcus aureus *and *Escherichia coli*

**DOI:** 10.1186/1753-6561-8-S4-P8

**Published:** 2014-10-01

**Authors:** Klebson Silva Santos, Ana Paula Barreto Prata Silva, Andriele Mendonça Barbosa, Isabelle Souza de Mélo Silva, Juliana Cordeiro Cardoso, Cláudio Dariva, Luiz Pereira da Costa, Malone Santos Pinheiro, Francine Ferreira Padilha

**Affiliations:** 1 Universidade Tiradentes, RENORBIO, Aracaju, 49032-490, Brazil; 2 Universidade Tiradentes, Campus Farolândia, Aracaju, 49032-490, Brazil

## Background

The treatment of bacterial diseases has become an increase public health problem, since the conventional antimicrobial substances lose their effectiveness due to the microbial resistance phenomenon [1]. Silver is a metal with potential antibacterial activity, and showed significant efficacy against several species of bacteria. The silver activity to prevent microbial growth is enhanced when the metal is found as nanoparticles [2]. Silver nanoparticles (NPAG) have their bacteriostatic and bactericidal activity based on their ability to kill or inhibit growth of bacteria. Pathogenic microorganisms, both gram positive and negative were sensitive to NPAG. The NPAG may be employed in conjunction with the biopolymers forming a nanocomposite that emerge as a promising therapeutic alternative. The xanthan gum is a biopolymer with interesting properties for food, cosmetic and pharmaceutical industries, acting as gelling and stabilizing [3]. The present study in focused on the evaluation of silver nanocomposite activity against gram positive and gram negative bacteria. The disk diffusion well technique was used to assess the efficiency of the silver nanocomposites against *Staphylococcus aureus *and *Escherichia coli*. The results indicated that the developed product has a broad spectrum of activity against the tested bacteria.

## Methods

The silver nanocomposite NAG was synthesized by *Xanthomonas *629, using silver nitrate in the fermentation media. The size of the nanoparticles was obtained by transmission electron microscopy. The metal concentration in the polymer was determined by atomic absorption spectrometric. The bacteria used in this study were *Staphylococcus aureus *and *Escherichia coli*. The evaluation of antimicrobial activity was assessed by the method of holes in wells, based on Mueller-Hinton agar. The abovementioned microorganisms were scattering from a microbial solution 10^8^, and the holes were subsequently filled with 50 µL of a silver nanoparticles (test ) solution and 50 µL of a xanthan gum alone (control) solution. All experiments were conducted in triplicate. An adaptation of the Kirby & Bauer technique using microbial suspension of 10^8 ^bacteria was also used for observing possible inhibition zones after incubation in bacteriological incubator at 37ºC for 24 hours.

## Results and conclusion

The analysis of transmission electron microscopy showed NAG about 3 ± 2 nm in size medium. The atomic absorption spectrometry showed a concentration of 49, 2 mg/g of metal per gram of NAG. The antimicrobial activity of silver NAG showed inhibition zones of 12.2 ** ± **0,3 mm and 11.6 ± 0,5 mm for Escherichia coli and Staphylococcus aureus, respectively. Both microorganisms were evaluated at a concentration of 5 mg NAG. The disk diffusion assay indicated that the sensitive zone of inhibition was 9.6 ± 0,4 mm and 9.7 ± 0,3 mm respectively for these pathogens, the control xanthan gum was not able of inhibit the growth of any pathogens tested. the results, it was concluded that the nanocomposite studied in this work presented a broad spectrum of antimicrobial activity, suggesting that use can be a viable alternative for the treatment of pathogenic organisms.
